# Antigenic and genotypic relatedness of buffalo-derived *Theileria parva* from Zambia to cattle-derived parasites and vaccine stocks

**DOI:** 10.1016/j.ijppaw.2025.101176

**Published:** 2025-12-10

**Authors:** Chimvwele Namantala Choopa, Walter Muleya, Lubembe Donald Mukolwe, Paul Fandamu, Kgomotso Penelope Sibeko-Matjila

**Affiliations:** a Department of Veterinary Tropical Diseases, Faculty of Veterinary Science, University of Pretoria, Private Bag X04, Onderstepoort, 0110 Pretoria, South Africa; b Central Veterinary Research Institute, Department of Veterinary Services, Ministry of Fisheries and Livestock, P. O. Box 33980, 10101 Lusaka, Zambia; c Department of Biomedical Sciences, School of Veterinary Medicine, University of Zambia, P. O. Box 32379, 10101, Lusaka, Zambia; d Department of Veterinary Pathology, Microbiology & Parasitology, Faculty of Veterinary Medicine and Surgery, Egerton University, P.O Box 536 Egerton, 20115, Kenya; e Department of Veterinary Services, Ministry of Fisheries and Livestock, P. O. Box 50060, 10101 Lusaka, Zambia

**Keywords:** Buffalo, Corridor disease, Antigenic diversity, Genetic diversity, Population structure, Microsatellite markers, Allelic profiles

## Abstract

The African buffalo (*Syncerus caffer*) is the natural reservoir of *Theileria parva*, a tick-transmitted protozoan parasite that severely constrains cattle production across eastern, southern, and central Africa. While the antigenic and genetic diversity of cattle-derived *T. parva* (causing East Coast fever) is well characterized in Zambia, little is known about the buffalo-derived parasites. The latter cause the equally fatal Corridor disease and impact the epidemiology and control of bovine theileriosis, particularly where live vaccines are used. This study investigated antigenic and genotypic diversity of *T. parva* from three Zambian buffalo populations, in comparison with cattle-derived parasites and vaccine stocks (Katete and Chitongo). Analysis of Tp1 and Tp2 antigen genes revealed contrasting diversity. Tp1 epitopes showed limited variation, whereas Tp2 exhibited extensive polymorphism, especially among buffalo-derived sequences. None of the variations have been previously reported in Zambia, suggesting ongoing diversification. Phylogenetic analysis showed paraphyletic clustering of buffalo and cattle parasites. However, some buffalo genotypes grouped closely with vaccine strains, suggesting potential cross-protection. Only two Tp2 sequences from buffalo-derived parasites clustered with those from cattle or vaccines, highlighting a risk of vaccine break-through. Population genetic analysis using multilocus genotyping demonstrated higher allelic richness and diversity in buffalo-derived parasites compared to cattle-derived. Although buffalo parasites showed greater multiplicity of infection (MOI) and unique alleles, principal coordinate analysis revealed limited genetic sub-structuring and shared alleles across hosts. These results suggest a common ancestry and overlapping transmission cycles. The greater MOI in buffalo populations highlights higher genetic exchange and can complicate control efforts. Overall, the study demonstrates extensive antigenic and genetic diversity of buffalo-derived *T. parva* in Zambia. These findings have critical implications for current vaccine efficacy, emphasizing the need for continuous molecular surveillance, strict livestock movement control, and vaccine efficacy trials to ensure effective management of *T. parva* infections.

## Highlights

• Buffalo are a major reservoir of *Theileria parva* antigenic and genetic diversity.
• Some Zambian buffalo-derived *T. parva* strains are related to ECF strains.
• Buffalo-derived *T. parva* genotypes shown a distant relationship to vaccine stocks.
• Current vaccines may offer limited protection against buffalo-derived *T. parva*.

## Graphical abstract

**Figure:**
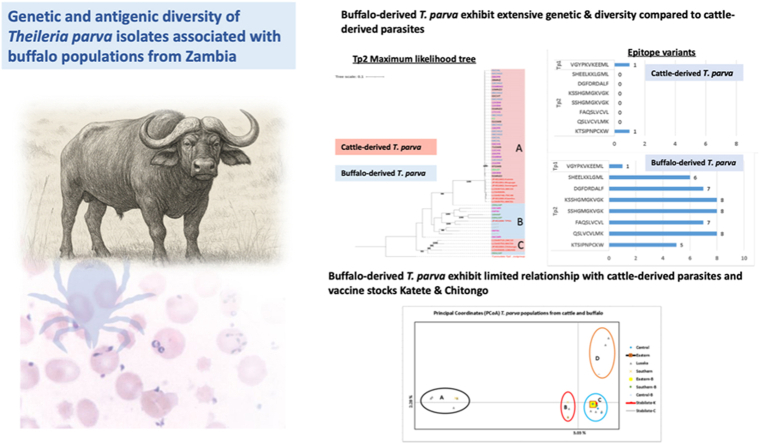

## 1 Introduction

Wildlife plays an important role in the epidemiology of several major livestock diseases, since it serves as reservoir host. Accordingly, the African buffalo (*Syncerus caffer*) is the natural reservoir of the protozoan parasite *Theileria parva* in East, Central and Southern Africa (Norval et al., 1992). The parasite is transmitted from buffalo to cattle by the brown ear-tick, *Rhipicephalus appendiculatus,* and causes bovine theileriosis. The disease is of economic importance due to high mortalities and the cost of control*.* Over time a subpopulation of *T. parva* has adapted to cattle and is maintained independently within this host. Tick-transmission of these parasites between infected and naïve cattle results in two distinct disease syndromes known as East Coast fever (ECF) and January disease (JD). Thus, the causative agents of these diseases are collectively referred to as ‘cattle-adapted’ or ‘cattle-derived’ *T. parva.* In contrast, parasites transmitted from buffalo to cattle, referred to as ‘buffalo-derived’ *T. parva*, cause a different disease syndrome known as Corridor disease.

East Coast fever is the form of bovine theileriosis endemic in Zambia. The first recorded case of ECF in the country occurred in 1922 at Fife in the Northern Province (Sempebwa-Serugo, 1977). In 1977/78, a malignant form of theileriosis was detected in the Hufwa area of Monze district in Zambia's Southern Province. This outbreak was subsequently diagnosed as Corridor disease (Nambota et al., 1994). Although Corridor disease is equally devastating to affected cattle populations, it has received limited attention in Zambia, as in many other countries where the disease co-occurs with ECF. Until recently, only one study has reported the detection of *T. parva* in buffalo in Zambia, based on serological analysis (Munang'andu et al., 2009). The first molecular detection and characterization of buffalo-derived *T. parva* was reported by Choopa et al. (2024).

The recent molecular detection of *T. parva* in selected buffalo populations in Zambia (Choopa et al., 2024) highlighted the ongoing risk of Corridor disease to cattle. In several regions of Zambia, unrestricted movement of buffalo increases the risk of transmission of buffalo-derived *T. parva* to cattle. As the principal tick vector *R*. *appendiculatus* is endemic in the country, parasite transmission can occur in areas where the two hosts co-graze. Immunisation of cattle by the infection and treatment method (ITM) remains the primary intervention for ECF in Zambia. In crossbred dairy cattle, ITM provides 97.6 % protection and reduces mortality by 97.9 % (Lynen et al., 2012). Two vaccine stabilates are used in Zambia: the Katete *T. parva* stock, applied in the Eastern Province, and the Chitongo stock, originally used in the Southern Province (Berkvens et al., 1988; Geysen et al., 1999). Since 2018, the use of the Chitongo stock has been expanded to Lusaka, Central and Copperbelt provinces, following the spread of ECF (AUDA-NEPAD, 2022). It remains unknown whether the current monovalent vaccines, Chitongo and Katete, can provide protection against buffalo-derived parasites. Therefore, the emergence and transmission of buffalo-derived *T. parva* genotypes that are genetically and antigenically different from those currently maintained in the cattle population could be devastating to the affected herds.

The efficacy of vaccine stocks depends on the antigenic profiles of the field parasites in cattle (Graham et al., 2006, 2008; Hemmink et al., 2016). Moreover, other studies have demonstrated that *T. parva* populations maintained in buffalo are genetically more diverse than those maintained in cattle (Allan et al., 2021; Geysen et al., 1999). This extensive diversity of *T. parva* populations in buffalo significantly complicates control of bovine theileriosis as recombination in the tick vector can give rise to novel parasite genotypes. The high diversity of buffalo-derived *T. parva* genotypes can be attributed to the buffalo's role as the parasite's ancestral host, having been exposed to a wider range of parasite strains over long evolutionary periods (Hemmink et al., 2016). Although monovalent vaccines are highly effective against cattle-derived parasites, they provide poor protection against the more diverse buffalo-derived *T. parva* parasites (Sitt et al., 2015). Even the trivalent vaccine Muguga cocktail, consisting of three parasite strains (Muguga, Kiambu and Serengeti), provides limited protection against diverse buffalo-derived *T. parva* parasites (Radley et al., 1979; Bishop et al., 2015).

Although the antigenic diversity and population genetics of *T. parva* have been extensively studied in populations maintained in cattle in Zambia (Muleya et al., 2012, 2022), data for parasites in the buffalo host remain scanty. MHC-I restricted CD8^+^ T-cells play a role in eliminating parasite-infected lymphoblasts and are crucial in the immune response against natural *T. parva* infections, and in cattle immunised with ITM (Taracha et al. (1995). Tp1 and Tp2 antigens have epitopes that are recognised by CD8^+^ T-cells (Graham et al., 2006, 2008; Hemmink et al., 2016) and have been shown to exhibit major sequence variations between different parasite isolates, even within the antigenic epitope regions (Pelle et al., 2011). As a result, these genes have been useful in studying the antigenic diversity of *T. parva* field populations from specific geographic areas, especially in preparation for introduction of ITM in new areas (Atuhaire et al., 2020, 2021; Salih et al., 2017).

Variable number tandem repeat (VNTR) loci, including micro- and minisatellite markers defined by short tandem repeats of 2–6 bp and 8–100 bp units respectively, have been widely used to study the genetic diversity and population structure of *T. parva* (Atuhaire et al., 2020; Lubembe et al., 2020; Muleya et al., 2012, 2022; Oura et al., 2003; Rukambile et al., 2016). These markers have been successfully used in studies of *T. parva* populations across several African countries, including Burundi (Atuhaire et al., 2021), Democratic Republic of Congo (Muleya et al., 2025), Kenya, Mozambique, South Africa (Katzer et al., 2010; Lubembe et al., 2020; Oura et al., 2005), Tanzania (Mwega et al., 2015; Rukambile et al., 2016), Malawi (Chatanga et al., 2020), Rwanda (Atuhaire et al., 2020) and Zambia (Muleya et al., 2012, 2022).

Thus, this study investigated the antigenic diversity of *T. parva* from selected buffalo populations in Zambia using two *T. parva* antigen genes, Tp1 and Tp2 (hereafter referred to as TpAg). We also assessed how antigenic signatures in buffalo-derived parasites relate to those of these TpAg in cattle-derived *T. parva* strains maintained in cattle and those used as vaccine stocks. Furthermore, we characterised the genotypes of *T. parva* populations from both cattle and buffalo, using a panel of minisatellite markers, to unravel the population structure of this parasite in Zambia for consideration in the control strategy against bovine theileriosis.

## 2 Methods

### 2.1 Ethical consideration

The study was approved by the Research and Animal Ethics Committees of the Faculty of Veterinary Science, University of Pretoria (REC Certificate # REC258–19) and the Central Veterinary Research Institute (CVRI), Zambia. Additional approvals were obtained from the Ministry of Fisheries and Livestock, Zambia, and the Department of Agriculture, Land Reform and Rural Development, South Africa. Approval for the use of archived blood samples from buffalo was obtained from CVRI.

### 2.2 Source of *Theileria parva* DNA

DNA samples used in this study originate from blood collected by Choopa et al. (2024). Whole blood samples from buffalo, collected from three provinces of Zambia (Central, Eastern and Southern) were obtained from the CVRI blood bank. Samples from cattle were collected from the same provinces, with an addition of one (Lusaka). DNA extraction and screening for *T. parva* DNA is described by Choopa et al. (2024). In total, 177 DNA samples positive for *T. parva* were used in this study, 43 from buffalo and 134 from cattle. Their distribution according to host and province of origin is shown in Table 1.

**Table 1:** Distribution of *Theileria parva* positive samples by host and geographic origin.

Geographic origin Province	Central		Eastern		Lusaka		Southern	
Sample Source	Cattle	Buffalo	Cattle	Buffalo	Cattle	Buffalo^a^	Cattle	Buffalo
**Number of *T. parva* positive samples**	40	10	42	16	35	n/a	27	7

a n/a- Buffalo blood and DNA samples from this province were not available.

### 2.3 Antigenic analysis of *Theileria parva* from cattle and buffalo

#### 2.3.1 PCR amplification of TpAg genes

DNA samples were used as template for PCR amplification of the 474 bp epitope-containing regions of Tp1 and Tp2 TpAg genes. The gene-specific primer sets, previously designed by Pelle et al. (2011), and PCR conditions used for amplification of the target regions in respective TpAg genes are shown in Table 2. The PCR reaction mixture for the amplification of each TpAg gene consisted of 1.25 μl 10X DreamTaq buffer (ThermoFisher Scientific™, Waltham MA, USA), 0.25 μl of 2 mM dNTPs, 0.25 μl of 10 μM of each forward and reverse primer, 0.0625 μl of 5 U/μl DreamTaq DNA polymerase (ThermoFisher Scientific™, Waltham MA, USA), 3 μl DNA template and nuclease-free water, in a total volume of 12.5 μl. The PCR products were assessed by gel electrophoresis on a 2 % GelRed-stained agarose gel.

**Table 2:** Primer sequences and annealing temperatures used for PCR amplification of *Theileria parva* antigen genes, Tp1 and Tp2, and the expected product sizes.

TpAg gene	Primer sequences (5′–3′)	Annealing temperature (°C)	Amplicon size (bp)
Tp1	forward-CTGGTGTACAATTTGGTGGG	50	428
	reverse-AACTTNMCTTCTTGCGAACC		
Tp2	forward-ATGAAATTGGCCGCCAGATTA	55	492
	reverse-AGATTTGTCACTAYCTGTWBYAGG		

#### 2.3.2 Sanger sequencing of TpAg gene amplicons

Tp1 and Tp2 amplicons were purified using Invitrogen™ PureLink™ PCR Purification Kit (ThermoFisher Scientific™, Waltham MA, USA) and their integrity assessed by gel electrophoresis on a 2 % GelRed-stained agarose gel. Purified amplicons were sequenced by bi-directional Sanger sequencing, using PCR primers for each respective TpAg gene (Table 2). The BigDye™ Terminator v3.1 Cycle Sequencing Kit (Applied Biosystems™, Waltham, MA, USA) was used to prepare sequencing reactions. Sequencing was performed on the ABI 3730XL DNA Sequencer (Applied Biosystems™, Waltham, MA, USA) for the samples that were processed in LGC Genomics GmbH (Germany), and on SeqStudio™ Genetic Analyzer (ThermoFisher Scientific, Waltham, MA, USA) for the samples processed at the University of Zambia (Lusaka, Zambia).

#### 2.3.3 Tp1 and Tp2 genes sequence and phylogenetic analyses

The quality of sequences for each TpAg gene was assessed using Trev from the Staden package (version 2.0.0b11-2016-windows-i386) (Staden et al., 2000). Confirmation of sequence similarities with respective to published sequences was performed using Basic Local Alignment Search Tool (BLAST) (https://www.ncbi.nlm.nih.gov/geo/query/blast.html). Pregap4 and Gap4 from the Staden package (Staden et al., 2000) were used for sequence assembly and editing of the consensus sequences. Subsequently, consensus sequences were aligned using the online version of Multiple Alignment Fast Fourier Transform (MAFFT) (Katoh et al., 2019). From cattle samples, 28 Tp1 and 34 Tp2 sequences were included in the analysis, and a total of 16 Tp1 and 11 Tp2 sequences from buffalo. Reference sequences used in this analysis are listed in Supplementary Table S1.

Prior to constructing the phylogenetic tree, the ModelFinder was used to determine the TpAg gene sequence dataset's best evolutionary model (Kalyaanamoorthy et al., 2017). The model TN + F + G4 was identified to be the best fit for Tp1 gene sequences and GTR + F + G4 for Tp2. Maximum likelihood phylogenetic trees were constructed based on the best models for each TpAg gene sequences with 1000 bootstrap iterations using ultrafast bootstrap (Hoang et al., 2018). Interactive Tree Of Life (iTOL) version 7 was used to visualise and annotate consensus trees using Newick files from IQ-Tree (Letunic and Bork, 2024). The trees were rooted using sequences of related genes from *T. annulata* (TA16450 for Tp1 and TA19865 for Tp2) as outgroups.

#### 2.3.4 Epitopes sequence variation analysis

Consensus sequences of TpAg genes were translated to predicted protein sequences using CLC Main Workbench 21.0.3 (Qiagen, Hilden, Germany). Tp1 and Tp2 predicted protein sequences encompassing the epitope regions were aligned with the respective reference sequences (Supplementary Table S1), using MAFFT version 7 (Katoh and Standley, 2013). The multiple sequence alignments were used to identify amino acid residue variations within the epitope regions of each TpAg.

#### 2.3.5 Haplotype analysis

All nucleotide sequences of TpAg genes used in the construction of phylogenetic trees, as well as reference sequences (Supplementary Table S1), were considered in the haplotype analysis using DnaSP v.6.12.03 (Rozas et al., 2017). The analysis was performed to determine the relatedness of *T. parva* field strains to the vaccine stocks. Subsequently, a median-joining (MJ) network was constructed for each TpAg gene sequences using Network Ver. 10 software (https://www.fluxus-technology.com/).

### 2.4 Genotyping and population structure analyses of *Theileria parva* from cattle and buffalo

#### 2.4.1 Amplification of selected minisatellite markers

Six minisatellite markers distributed on four chromosomes of the *T. parva* genome (Table 3), described by Oura et al. (2003), were selected for genotyping buffalo- and cattle-derived parasites from Zambia. Specific primer sets (Oura et al., 2003), each including a fluorescent labeled forward primer, were used for PCR amplification of each locus. A 12.5 μl PCR reaction mix used comprised of 1.25 μl 10X DreamTaq buffer (ThermoFisher Scientific™, Waltham, MA, USA), 0.25 μl of 2 mM dNTPs, 0.25 μl of 10 μM of each primer, 0.0625 μl of 5 U/μl DreamTaq DNA polymerase (ThermoFisher Scientific™, Waltham, MA, USA), 3 μl of 10 ng DNA template and nuclease-free water. The PCR conditions consisted of initial denaturation at 95 °C for 3 min, 35 cycles including denaturation at 95 °C for 15 s, annealing at 60 °C for 30 s and extension at 72 °C for 1 min, followed by a final extension step at 72 °C for 15 min. The amplicons were analysed on 2 % agarose gels stained with ethidium bromide, in a 1X Tris–acetate– EDTA buffer and the DNA bands were visualised under UV light.

**Table 3:** Satellite markers used to genotype *Theileria parva* parasites from buffalo and cattle from selected provinces in Zambia.

^#^Marker	Chromosome	Amplicon size range (bp)
**MS7**	**1**	150–380
**MS8**	**1**	160–330
**MS19**	**2**	130–320
**MS25**	**3**	180–340
**MS33**	**4**	150–220
**MS39**	**4**	230–420

#### 2.4.2 Capillary flow genotyping of *Theileria parva* DNA

Minisatellite amplicons were prepared for capillary electrophoresis as described by Patel et al. (2011). Reaction mixtures comprising 1 μl PCR product, 9 μl of Hidi formamide and 0.4 μl GeneScan™ 600 LIZ™ Size Standard v2.0 (Applied Biosystems™, ThermoFisher Scientific™, Waltham, MA, USA) were denatured at 95 °C for 6 min in a GenAmp PCR system 9700 thermocycler. The denatured PCR products were separated by capillary electrophoresis in an ABI 3500 SeqStudio Genetic Analyzer (Applied Biosystems®, ThermoFisher Scientific™, Waltham, MA, USA). Gene Mapper™ Software ver. 5 (Applied Biosystems™, ThermoFisher Scientific™, Waltham, MA, USA) was used to analyse the DNA fragment sizes relative to the ROX-labeled GS600 LIZ size standard. For each sample, the alleles with the highest peaks and wide area under the curve (quantitative measurement) were considered as predominant. Alleles that measured to at least one third of the predominant height were considered as minor alleles (Salih et al., 2018). The data from the predominant alleles was combined to produce multi-locus genotypes (MLG). Only alleles with products of sizes correlating with the prescribed base pair range (Table 3) were considered in generating the MLG.

#### 2.4.3 Population genetic diversity analysis

For the analysis of the level of similarity among the MLG, the microsatellite tool kit (http://animalgenomics.ucd.i.e.,/sdepark/ms-toolkit/) was utilised. The allele frequency distribution was analysed using the principal coordinate analysis (PCoA). Thereafter, the results were visualised utilising Genalex 6 (Peakall and Smouse, 2006). Arlequin version 3.5 (Excoffier et al., 2007) was used for the Analysis of Molecular Variance (AMOVA) and evaluation of the extent of differentiation in the population. To assess the null hypothesis of panmixia and linkage equilibrium among the study populations, Linkage Analysis (LIAN) (version 3.7) (http://guanine.evolbio.mpg.de/cgi-bin/lian/lian.cgi.pl) was used (Haubold and Hudson, 2000). LIAN calculates standardized index of association (*I*_*A*_^*S*^), variance of pairwise differences (V_D_), variance of differences required for panmixia (V_E_) and the 95 % confidence interval (L). Where *T. parva* populations were freely mating, the *I*_*A*_^*S*^ value was negative or close to zero. *Theileria parva* populations were non-panmictic when *I*_*A*_^*S*^ values was positive or greater than zero. The null hypothesis of panmixia and linkage disequilibrium (LD) was rejected when V_D_ was greater than L. LIAN v3.7 was also used to determine multiplicity of infection (MOI).

## 3 Results

### 3.1 Antigenic diversity of *Theileria parva* from cattle and buffalo

#### 3.1.1 Phylogenetic relationship between TpAg gene sequences from cattle- and buffalo-derived *T. parva*

A total of 131 PCR products were obtained from amplification of Tp1 and Tp2 genes from *T. parva* positive DNA from buffalo and cattle samples. The number of sequences obtained from amplicons of each TpAg gene are shown in Table 4. Overall, 44 Tp1 gene sequences were obtained from DNA from 30 cattle and 12 buffalo samples. For the Tp2 gene, 45 sequences were obtained from DNA from 34 cattle and 11 buffalo samples.

**Table 4:** Number of DNA sequences obtained from cattle and buffalo samples, from different provinces in Zambia.

*T. parva* antigen gene	Sequences obtained per host from different provinces of Zambia									
	Central		Eastern		Lusaka	Southern		Sub-total		Total
	Cattle	Buffalo	Cattle	Buffalo	Cattle	Cattle	Buffalo	Cattle	Buffalo	
**Tp1**	8	4	2	8	12	6	4	28	16	44
**Tp2**	12	4	2	4	12	8	3	34	11	45

**Fig. 1:**
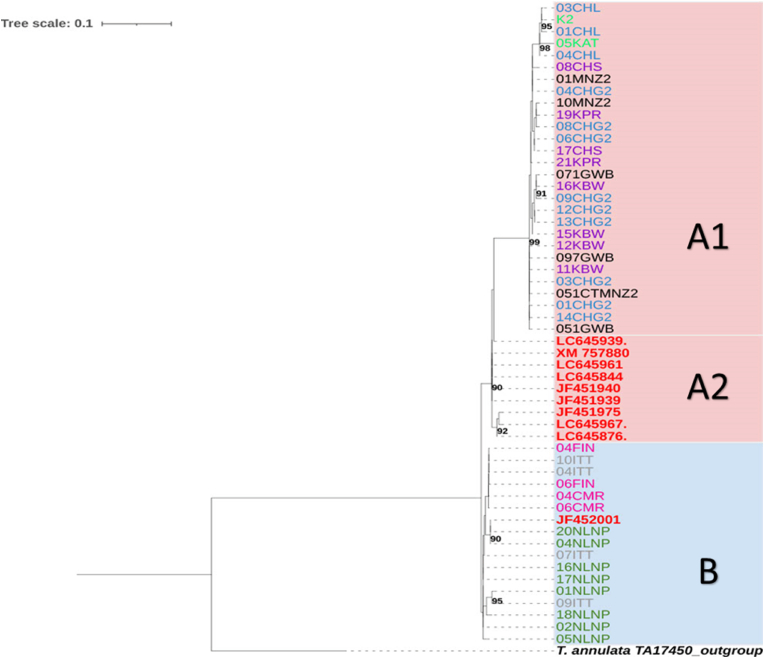
*Theileria parva* Tp1 antigen gene Maximum-likelihood phylogenetic tree. A total of 55 nucleotides sequences representing different cattle (n = 28) and buffalo (n = 16) samples, as well as references (n = 10) and an outgroup, were analysed with 1000 bootstrap iterations using Ultrafast bootstrap in IQ-Tree. The final dataset consisted of a total of 325 positions. The salmon pink background highlights sequences from cattle and the blue, sequences from buffalo samples. Sequence names for cattle samples from Central, Eastern, Lusaka and Southern provinces are shown in purple, green, blue and black font, respectively. Sequences from buffalo samples from Central, Eastern and Southern provinces are shown in pink, olive green and grey font, respectively. Reference sequences are shown in red font including Chitongo (JF451975), Katete (LC645844), Muguga (XM_757880), Kiambu 5 (JF451939) and Serengeti (JF451940) vaccine stocks. The outgroup is shown in bold and italics black font.

The Tp1 gene phylogenetic tree revealed two major clades, A and B (Fig. 1), although without bootstrap support. This observation is consistent with insufficient informative character variation in these sequences. Clade A (highlighted in pink background) recovered two subclades: A1 containing sequences from cattle in the Central, Eastern, Lusaka and Southern provinces, and A2 containing reference sequences from cattle-derived *T. parva,* including vaccine stocks from Zambia [Chitongo (accession number: JF451975) and Katete (LC645844)] and Kenya [Muguga (XM_757880), Kiambu 5 (JF451939) and Serengeti (JF451940)]. Clade B (highlighted in blue) exclusively consisted of sequences from buffalo, representing all the provinces where buffalo samples originated together with a buffalo reference sequence (JF452001) from Kenya.

**Fig. 2:**
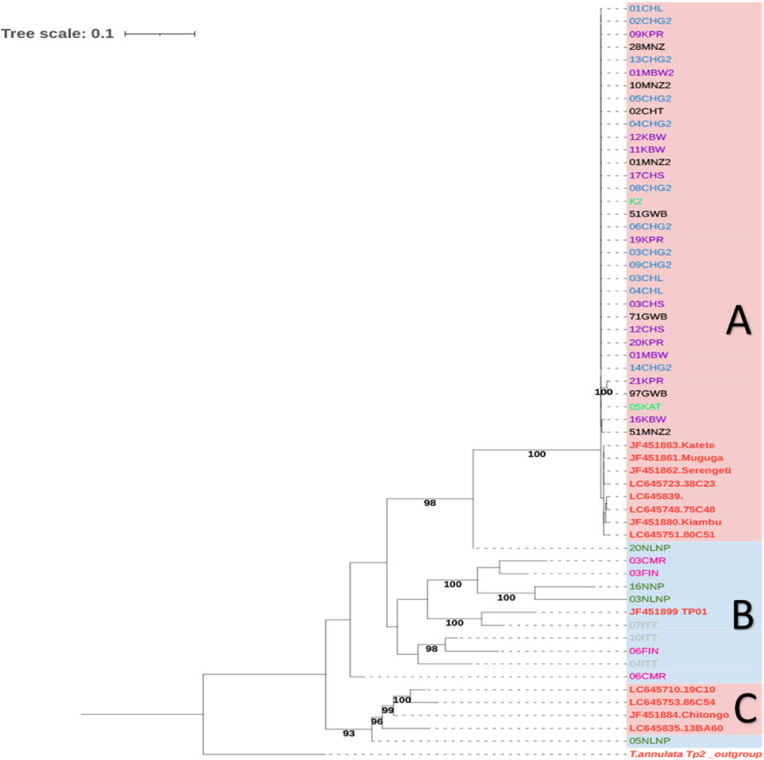
*Theileria parva* Tp2 antigen gene Maximum-likelihood phylogenetic tree. A total of 59 nucleotides sequences representing different cattle (n = 34) and buffalo (n = 11) samples, as well as references (n = 13) and an outgroup, were analysed with 1000 bootstrap iterations using Ultrafast bootstrap in IQ-Tree. The final dataset comprised of 474 base pairs. The salmon pink background highlights sequences from cattle and the blue, sequences from buffalo samples. Sequences from cattle samples from Central, Eastern, Lusaka and Southern provinces are shown in purple, green, blue and black font, respectively. Sequence names for buffalo samples from Central, Eastern and Southern provinces are shown in pink, olive and grey font, respectively. Reference sequences are shown in red font including Chitongo (accession number: JF451884), Katete (accession number: JF451863), Muguga (accession number: JF451861), Kiambu 5 (accession number: JF451880) and Serengeti (accession number: JF451862) vaccine stocks. The outgroup is shown in bold and italics red font.

The Tp2 gene phylogenetic tree showed three distinct clades, A, B and C (Fig. 2). Clades A and C contained sequences associated with cattle-derived parasites (highlighted in pink). Notably, all sequences from cattle field samples grouped together in cluster A, with one sequence from buffalo from the Eastern Province. Although in the same clade with sequences from cattle-derived parasites, the latter sequence had a separate branch from the rest of the sequences in this clade. Clade C consisted of the sequence from the vaccine stock, Chitongo, and three other reference sequences from cattle-derived parasites from Central and Lusaka provinces (Muleya et al., 2022). Notably, a single sequence from a buffalo-derived parasites from the Eastern Province was also found in this clade. On the other hand, clade B exclusively constituted sequences from buffalo-derived parasites, including a reference sequence of a strain from Kenya (JF451899). Overall, there was no geographical segregation among Tp2 gene sequences from both cattle and buffalo.

#### *3.1.2* Detection of epitope variants in Tp1 and Tp2 antigens predicted sequences

**Fig. 3:**
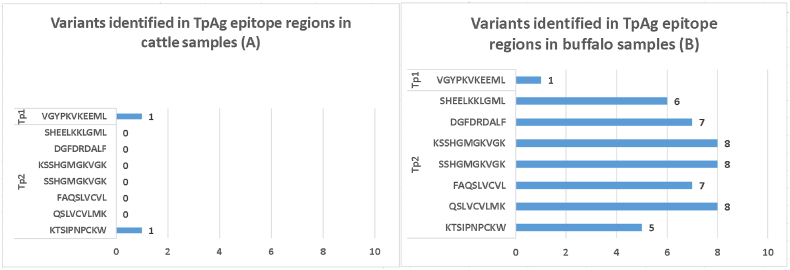
Number of epitope variants identified in Tp1 and Tp2 gene sequences from *Theileria parva* parasites from cattle (A) and buffalo (B).

The cattle-derived Muguga sequences were used as references to identify variants at the antigenic epitope regions of Tp1 and Tp2 sequences obtained from *T. parva* from buffalo and cattle from Zambia (Fig. 3; Table 5). Overall, two epitope variants were detected from the two Tp1 epitope regions, from the 42 sequences analysed. In contrast, 49 epitope variants were identified from the seven Tp2 epitope regions, from 39 sequences analysed (Fig. 3; Table 5). Notably, one Tp1 and 49 Tp2 epitope variants were detected in sequences from parasites from the buffalo. Further, the Tp1_214-224_ (VGYPKVKEEII) and Tp2_138-147_ (KEDIPNPCKW) epitope variants were common in both buffalo and cattle (Table 5). Tp2 epitope regions were the most variable, with up to eight amino acid residue substitutions per epitope region. On the other hand, the Tp1 epitope region in sequences from buffalo-derived parasites had two variants while only one variant was detected in sequences from cattle-derived *T. parva*.

**Table 5:** Epitope variants identified from *Theileria parva* antigens, Tp1 and Tp2, of parasites from cattle and buffalo.

Sample	Tp1 epitope variants		Tp2 epitope variants													
	aNo.	Tp1_214-224_	No.	Tp2_27-37_	No.	Tp2_40-48_	No.	Tp2_49-59_	No.	Tp2_50-59_	No.	Tp2_96-104_	No.	Tp2_98-106_	No.	Tp2_138-147_
Muguga reference		VGYPKVKEEML		SHEELKKLGML		DGFDRDALF		KSSHGMGKVGK		SSHGMGKVGK		FAQSLVCVL		QSLVCVLMK		KTSIPNPCKW
Cattle epitope variant(s)	30	^#^VGYPKVKEE**II** (6)	29	∗∗None	30	∗∗None	30	∗∗None	30	∗∗None	30	∗∗None	30	∗∗None	29	^#^K**ED**IPNPCKW (1)


Buffalo epitope variant(s)	12	VGYPKVKEEM**I** (1)	6	S**DD**EL**DT**LGML (1)	7	**E**GF**EKEK**LF (1)	8	**LT**S**K**GMA**T**VG**R** (1)	8	**T**S**K**GMA**T**VG**R** (1)	9	FAQS**IQ**CV**S** (1)	9	QS**IQ**CV**SQH** (1)	5	K**VMF**PNP**LSN (1)**
		^#^VGYPKVKEE**II** (1)		S**D**EE**LESL**GML (1)		DG**L**D**K**D**E**LF (1)		KSS**KA**M**TTT**GK (1)		SS**KA**M**TTT**GK (1)		F**G**QS**IK**CV**V** (2)		QS**IK**CV**VQ**K (2)		^#^K**ED**IPNPCKW (1)
				**TD**EELK**N**LGML (1)		**PDL**D**KNR**LF (1)		K**T**S**KA**M**TTT**G**R** (1)		**T**S**KA**M**TTT**G**R** (1)		FAQS**IK**CV**V** (1)		QS**IK**CV**VK**K (1)		K**P**S**V**PNPC**D**W (1)
				S**D**EELKKLGML (1)		**PNP**D**KEK**LF (1)		**LT**SHGMG**RI**GK (1)		**T**SHGMG**RI**GK (1)		FA**A**S**IK**CV**A** (1)		**A**S**IK**CV**AQY** (1)		K**DNT**PNPCKW (1)
				S**DD**ELKK**M**GM**I** (1)		**PVS**D**KEK**LF (1)		**LT**S**KA**M**TT**VGK (1)		**T**S**KA**M**TT**VGK (1)		FAQS**IK**CV**A** (2)		Q**SIK**CV**AHH** (1)		K**QDV**PNP**CE**W (1)
				S**DD**ELKK**M**G**LI** (1)		DG**Q**D**SLTRK** (1)		**LT**S**K**GM**TT**VG**R** (1)		**T**S**K**GM**TT**VG**R** (1)		**LVRIV**L**N**VL (1)		QS**IK**CV**AQN** (1)		
						**H**GFD**KEV**LF (1)		KSS**KA**M**TTT**G**R** (1)		SS**KA**M**TTT**G**R** (1)		**TGV**S**EP**C**LV** (1)		**RIVLN**VL**H**K (1)		
								KS**MELDLEQQ**K (1)		S**MELDLEQQ**K (1)				**V**S**EP**CLV**CF** (1)		

Total number	42		35		37		38		38		39		39		34	

a No. = total number of sequences analysed; ∗∗None = no variants detected; # = Variants found in both buffalo and cattle; () = Number of sequences from which the variant was detected.

#### 3.1.3 Relatedness of *Theileria parva* field and vaccine stocks

**Fig. 4:**
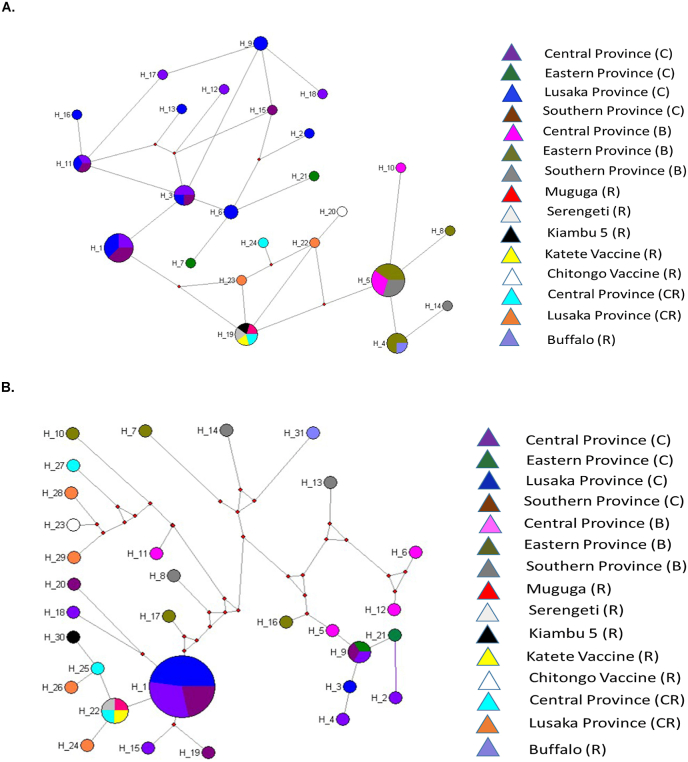
Media-joining network of *Theileria parva***(A)** Tp1 and **(B)** Tp2 antigen gene sequences constructed using Network 10, based on polymorphic sites*.* The size of circles denotes the haplotype frequency while the branches gives an idea of the relationship between and among samples. Colour codes indicate the origin of the samples. C = cattle, B = Buffalo, R = vaccine stock reference sequence, CR = Zambian cattle reference sequence.

The analysis of 54 Tp1 nucleotide sequences by MJ network revealed 24 haplotypes (Fig. 4A). Haplotype H5 (n = 10) was the most common and contained buffalo sequences from all three populations (Central, Eastern and Southern provinces). Notably, all haplotypes radiate from H5. The next common was haplotype H1 comprising of sequences from cattle-derived strains (n = 8). Haplotype H19 (n = 5) followed with reference sequences including vaccine stocks, Muguga, Serengeti, Kiambu 5 and Katete, and sequences of cattle-derived strains from Central Province, Zambia (Muleya et al., 2022). Interestingly, the Chitongo vaccine stock was the only sequence in H20, and was linked to the other vaccine haplotype (H19) through H22 consisting of a cattle reference sequence from Lusaka Province. Other sequences associated with parasites from buffalo formed part of haplotype H4, especially from Eastern Province (n = 3) and a reference buffalo sequence (JF452001) from Kenya. The rest of the haplotypes consisted of sequences from cattle-derived parasite populations, with multiple geographic origin.

The MJ network analysis for Tp2 gene sequences identified 31 haplotypes (Fig. 4B). Three major haplotypes were identified, H1, H22 and H9, and all consisted of sequences from cattle samples. The most frequent haplotype was H1 (n = 23), consisting of sequences originating from cattle from Central, Lusaka and Southern provinces, with the majority coming from Lusaka (n = 11). Haplotype H22 (n = 4) comprised of three of five reference sequences from vaccine stocks (Muguga, Serengeti and Katete), and a sequence from cattle from Central Province. H23 and H30 haplotypes also contained reference sequences for Chitongo and Kiambu 5 vaccine stocks, respectively. Notably, haplotype H30 was linked to the other vaccine haplotype (H22) through H25 consisting of a cattle sequence from Central Province. The third major haplotype, H9, consisted of three cattle-derived sequences from three different provinces. The rest of the network (25 haplotypes) comprised of single sequence haplotypes. Interestingly, 11 of these consist of all sequences from buffalo associated parasites, with sequences from Eastern Province in H7, H10, H16 and H17; Central Province in H5, H6, H11 and H12, and Southern Province in H8, H13 and H4 haplotypes. Notably, all these haplotypes, except one (H5), are indirectly linked to others, consisting of references (including vaccine stocks) and cattle-derived sequences, through a vector.

### 3.2 Genotypic diversity of *Theileria parva* from cattle and buffalo

#### 3.2.1 Satellite loci diversity, allele frequencies and predominant alleles

**Fig. 5:**
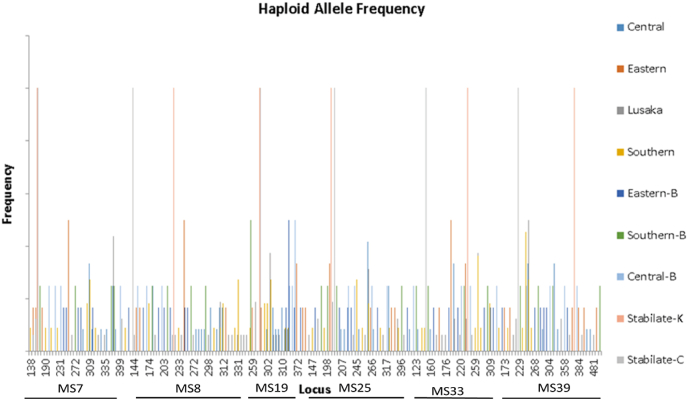
The overall allele frequencies in *Theileria parva* vaccine strains and field parasites from cattle and buffalo originating from Central, Eastern, Lusaka and Southern provinces of Zambia. The histograms were generated using multi-locus genotype (MLG) data. Vaccine stabilate-K = Katete and stabilate-C = Chitongo. Parasite populations from cattle samples are listed as Central, Eastern, Lusaka and Southern while Central-B, Eastern-B and Southern-B represent parasite populations from buffalo.

The PCR amplification of selected satellite loci (n = 6) from *T. parva* positive buffalo and cattle DNA samples, produced 78 amplicons. The satellite loci analysis showed great polymorphism among the *T. parva* parasites from both cattle and buffalo. MS8 was the most polymorphic (Na = 50) minisatellite while MS19, M33 and M39 (Na = 40) were the least (Table 6). The unbiased diversity (uh) of alleles from parasites associated with buffalo was higher, ranging from 0.944 to 0.972, compared to 0.851 to 0.885 for those from cattle. This indicates a more diverse *T. parva* population in buffalo. Allele frequencies showed high proportion of specific alleles for each locus within different populations (Fig. 5). They ranged from as low as 0.06 in parasite populations from cattle and 0.17 in buffalo associated populations, to 1 for both populations (Supplementary Table S2).

**Table 6:** Alleles detected from *Theileria parva* from cattle and buffalo in provinces of Zambia.

Population by Province	N	MS7	MS8	MS19	MS25	MS33	MS39	uh	Total Na per population
^#^**Cattle samples (Na)**									
Central	12	7	9	**9**	6	5	6	0.859	42
Eastern	6	4	4	5	5	3	6	0.867	27
Lusaka	16	**8**	**14**	8	**8**	**9**	**7**	0.851	54
Southern	11	**8**	7	7	**8**	7	5	0.885	42
^#^**Buffalo samples (Na)**									
Eastern-B	6	**6**	**6**	**4**	**6**	**6**	**6**	0.967	34
Southern-B	4	4	4	2	4	4	4	0.944	22
Central-B	4	4	4	3	4	4	4	0.972	23
**Vaccine stabilates (Na)**									
Katete	1	1	1	1	1	1	1	0.000	6
Chitongo	1	1	1	1	1	1	1	0.000	6

Total Na per loci		43	50	40	43	40	40		256
Mean uh		0.711	0.738	0.66	0.727	0.691	0.704	0.705	

N = sample size, uh = unbiased diversity = (N/(N-1)) h, Na = number of different alleles, Numbers in bold indicate the highest number of alleles identified per loci, per host of origin.

*Theileria parva* populations from cattle recorded more shared alleles at all loci, but one (MS39), compared to those from buffalo (Supplementary Table S3). Notably, locus MS19 showed the highest number of shared alleles (n = 8) between cattle-derived parasite populations while only two loci (MS8 and MS19) had shared alleles between parasite populations from the buffalo. Comparison across parasite populations from buffalo and cattle showed shared alleles in all loci, with most alleles detected in MS8 and MS19.

Generally, predominant alleles were identified in all cattle-derived parasite populations, in all loci, except for MS39 in Eastern Province population (Supplementary Table S4). Notably, the cattle-derived *T. parva* populations from the Central Province and buffalo-derived parasites from the Southern Province had the most MLGs, three and two respectively, while the rest of the populations had single MGLs.

#### 3.2.2 Multiplicity of infections (MOI)

*Theileria parva* populations from cattle (n = 45) and buffalo (n = 14) comprised of multiple genotypes. The MOI mean index was measured at each locus within cattle and buffalo *T. parva* populations. The parasite populations from buffalo showed greater genotype co-infection, recording higher minimum and maximum mean values of 1.57 and 3.43 respectively. In contract, parasite populations from cattle recorded lower minimum (1.11) and maximum (1.84) mean values. Consistently, *T. parva* populations from buffalo had a higher overall mean (2.24) and standard deviation (0.66), even higher than the overall mean and standard deviation for both parasite populations (cattle and buffalo), which were 1.80 and 0.32, respectively.

#### 3.2.3 Similarity analysis between *Theileria parva* parasites buffalo and cattle populations

**Fig. 6:**
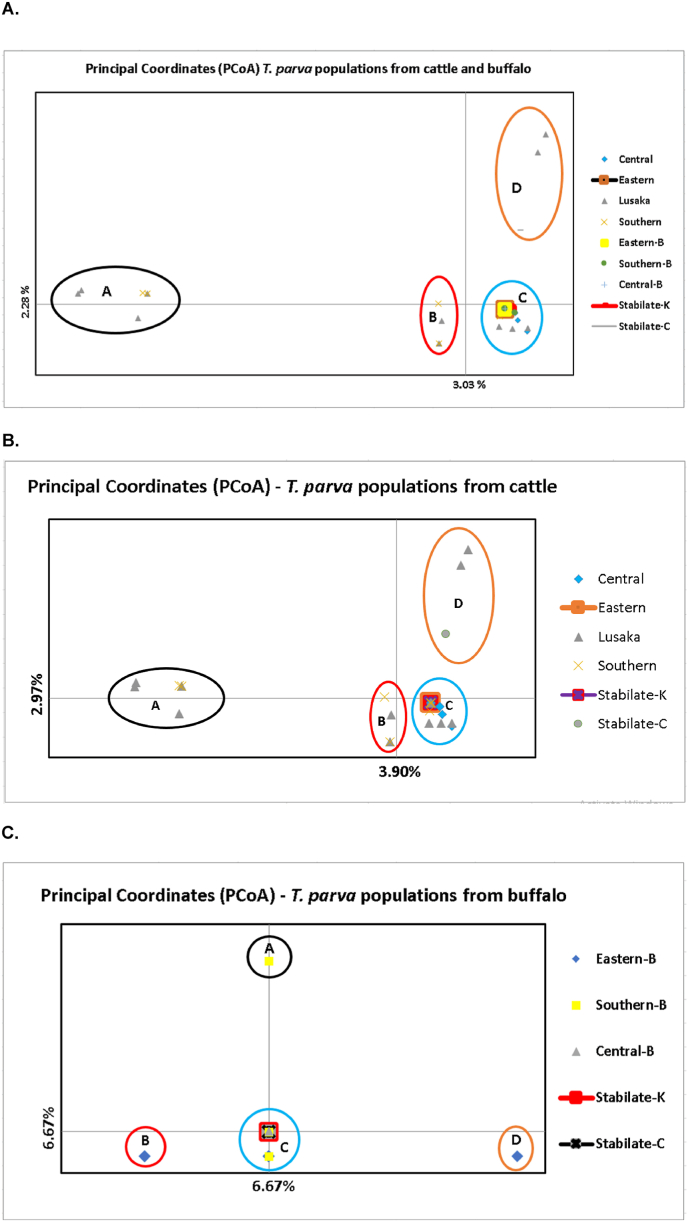
Principal coordinate analysis (PCoA) of *Theileria parva* populations from (A) cattle, buffalo and vaccine strains Chitongo and Katete; (B) cattle and vaccine strains; (C) Buffalo and vaccine strains. Cattle samples originate from Central, Eastern, Lusaka and Southern provinces, which buffalo are from Eastern, Central and Southern provinces. PCoA was performed using multi-locus genotype data.

Analysis of molecular variance (AMOVA) showed that all variations occurred among parasites within individual populations (100 %). The PCoA revealed the distribution of genotypes from cattle and buffalo-derived *T. parva* parasites into four quadrants (Fig. 6A). All genotypes from buffalo formed part of cluster C, together with some genotypes from cattle-derived parasites from Central, Eastern, Lusaka and Southern provinces. Notably, *T. parva* genotypes from Lusaka cattle were spread in all four clusters (A, B, C and D). A close relationship between genotypes of parasites from cattle from Southern Province and Lusaka was observed in clusters A, B and C. Genotypes representing the Chitongo vaccine stabilate grouped with those from cattle from Lusaka in cluster D. In cluster C, Katete showed a close genetic relationship with genotypes from cattle from all four provinces under investigation. The proportion of the total variance between clusters A, B and C was noticeably low at 2.28 % and against cluster D at 3.03 %.

A separate PCoA of *T. parva* parasite genotypes from cattle and vaccine stabilates (Fig. 6B) showed the clustering similar to that including genotypes from both cattle- and buffalo-derived parasite populations (Fig. 6A). Similarly, the proportion of the total variance for each coordinate was low at 2.97 % and 3.90 % for the x- and y-axes respectively. The PCoA for *T. parva* genotypes from buffalo and vaccine stabilates (Fig. 6C) revealed higher diversity, especially for populations from Eastern and Southern provinces, distributed in more than one cluster. Consistently, the proportion of the total variance for each coordinate was higher at 6.67 % in both the x- and y-axes. Additionally, most genotypes from the Eastern Province clustered separate from those from Central and Southern provinces. All buffalo-derived parasite populations had genotypes that clustered with vaccine genotypes in cluster C.

#### 3.2.4 Inter and intra linkage of *Theileria parva* populations

Evaluation of linkage among and within individual *T. parva* populations was performed using allelic profile information obtained from the four immunising provinces of Zambia. When all populations were analysed as a single population, a standard index of association (*I*_A_^S^) of 0.0453 (Supplementary Table S5) was observed, indicating a non-panmictic population. When the observed mismatch value (V_D_) and critical value (L_MC_) were compared, V_D_ (1.9647) was greater than L_MC_ (1.6040) (Supplementary Table S5), indicating that the combined populations were in a state of linkage disequilibrium. It was also noted that all individual populations had standard index of association values that were greater than zero and mismatch values greater than critical values, indicating that all the individual populations were in linkage disequilibrium (LD).

## 4 Discussion

The ITM has been implemented in eastern and parts of southern Africa, including Zambia, as an affordable alternative to chemotherapy for control of bovine theileriosis (Patel et al., 2016; Muleya et al., 2022). However, pathogens with different antigenic profiles can evade the immune responses developed against other strains (Gomes et al., 2002; Lipsitch and O’Hagan, 2007). This cycle of immune response and pathogen evasion leads to a diverse population of pathogen strains, each with distinct antigenic characteristics. As natural reservoir hosts for *T. parva*, buffalo play a central role in the diversification of the parasite genotypes and antigenicity. In this context, this study investigated the antigenic and genotypic diversity of buffalo-derived *T. parva* in comparison with parasites from cattle across four provinces of Zambia and the current vaccine stocks used in ITM.

### 4.1 Antigenic diversity in buffalo-derived *Theileria parva* and relatedness to vaccine strains

Analysis of the two TpAg genes and their predicted protein sequences showed contrasting results, with Tp1 revealing limited variation compared to Tp2, particularly among parasites associated with buffalo. This is consistent with earlier studies that reported extensive genetic and antigenic diversity among buffalo-derived *T. parva* populations (Lubembe et al., 2021; Muleya et al., 2022; Pelle et al., 2011; Salih et al., 2017; Sitt et al., 2018). The greater diversity observed in the Tp2 sequences may be due to the higher number of antigenic epitopes in Tp2 (n = 7) compared to Tp1 (n = 1) (Graham et al., 2008; Nene et al., 2012). It further suggests that this antigen is highly prone to mutation, which is common for parasite evasion of the host response. Consistent with this, the amino acid variants in Tp2 sequences from buffalo-derived parasites ranged from five to eight per epitope. Interestingly, there was only a single amino acid variant detected from cattle-derived parasite sequences. As the host develops an immune response to parasite's dominant antigens, the parasite population experiences selective pressure to present different antigens to avoid being eliminated (Frank, 2002). Considering that buffalo is the ancestral host of *T. parva* (Norval et al., 1991)*,* it is possible that in this host, antigenic variation results in persistent infections while in cattle it leads to chronic infection due to varying host responses. Notably, none of the variants matched those identified in earlier Zambian studies (Muleya et al., 2022), highlighting the dynamic nature of field *T. parva* populations.

Phylogenetic analyses of Tp1 and Tp2 showed a paraphyletic relationship between sequences from parasites associated with buffalo and cattle, with each group forming distinct clades. This is consistent with the fact that *T. parva* parasites share a common ancestor, which originates from the natural reservoir host, the buffalo. The segregation of sequences from cattle- and buffalo-derived parasites was well supported for the polymorphic Tp2 and revealed a close relationship of two sequences from buffalo-derived parasites with those from cattle-derived parasites. Each of the sequences from buffalo-derived parasites forms part of distinct clades containing sequences from the vaccine stocks used for immunisation of cattle against *T. parva* infections in Zambia, Katete and Chitongo. Consistently, Tp2 haplotypes for the vaccine genotypes, Katete (H22) and Chitongo (H23), and buffalo-derived parasite genotypes shared a distant but common origin (H1). This suggests that immunisation with cattle-derived *T. parva* vaccines stocks currently in use in Zambia may offer protection against infections with some of the buffalo-derived parasites.

### 4.2 Genotypic diversity and population structure

The population genetic analysis showed that buffalo-derived *T. parva* parasite populations from the Eastern Province had the highest number of different alleles in all loci compared to the other two populations, indicating greater genetic diversity. Of the estimated 40,000 to 50,000 buffalo in Zambia (https://www.expertafrica.com/wildlife/buffalo/zambia), more than 30,000 occur in the Luangwa ecosystem in Eastern Province and the remainder is distributed across other national parks. This large buffalo population likely contributes to the extensive diversity observed in the region's *T. parva* parasites. The analysis of alleles from parasite populations from the Eastern Province, where the sample size was the same for both cattle and buffalo, showed that buffalo-derived parasites had higher allele numbers in all loci compared to those from cattle, with total Na of 34 and 27 respectively. In pathogen populations, the number of alleles per locus is directly linked to the pathogen's genetic diversity. A higher number of alleles at a locus means there are more variations in the genetic makeup of the pathogen population. These findings suggest that the diversity among parasite genotypes from buffalo is more extensive than those in cattle. This is further supported by the high unbiased diversity values recorded for buffalo-derived parasite populations, which ranged between 0.944 and 0.972, compared to 0.851–0.885 for cattle-derived parasites. These patterns well align with previous studies from Zambia reporting limited genetic diversity among *T. parva* populations maintained in cattle in some provinces (Muleya et al., 2012, 2022).

Allele frequency analysis in this study showed high proportion of locus-specific alleles within buffalo- and cattle-derived parasites. Although both buffalo- and cattle-derived *T. parva* populations had several shared alleles on the different loci within each population, few loci with shared alleles were detected from genotypes associated with populations from buffalo (MS8 and M19), possibly due to a small sample size. Interestingly, when alleles from both parasite populations (cattle and buffalo) were analysed together, shared alleles were observed at all loci, suggesting a common ancestral origin for the two populations and possibly, minimal genetic sub-structuring. This is further supported by the close relationship between buffalo-derived genotypes and some cattle-derived genotypes observed in cluster C of the PCoA. The limited genetic sub-structuring between the parasite populations from cattle and buffalo could be an advantage from the control perspective. It could mean that infections with some of the buffalo-derived parasites can be controlled with the current vaccines used for ITM in Zambia, a hypothesis that requires further investigation. However, AMOVA showed that all variations occurred among parasites within individual populations. Consistently, unique alleles exclusive to sub-populations of parasites associated with cattle and buffalo were also detected. These alleles could be explored as potential tools for tracking parasite populations during outbreaks, monitoring geographic spread, and understanding transmission dynamics over time.

The MLG quantitative method was also used to define the structure of *T. parva* parasites from cattle, buffalo and vaccine stocks from Zambia. Although the selection of predominant alleles has its limitations, the method is currently considered a reliable standard for characterising *T. parva* parasites (Atuhaire et al., 2021; Muleya et al., 2012; Oura et al., 2005; Simuunza et al., 2011). Analysis of predominant alleles revealed a low number of MLGs across both cattle- and buffalo-derived parasite populations, generally a single MLG, suggesting low genetic diversity. Low MLG diversity can be attributed to several factors including population-level interventions like mass drug treatments or other intervention packages; reduction in the potential for sexual reproduction and outcrossing; low host density and geographic isolation such as low-transmission setting (Daniels et al., 2020). Although a combination of these could apply to Zambia, one likely scenario for the population analysed in this study could be geographic isolation. Linkage analysis revealed that there is linkage disequilibrium (LD) among *T. parva* populations from the buffalo and cattle from Zambia. The standard index of association for combined populations indicated a non-panmictic state, most likely due to the geographic separation of the study populations, making possible interactions of the hosts unlikely. Although this phenomenon is common to both buffalo- and cattle-derived *T. parva* parasites (Muwanika et al., 2016; Odongo et al., 2006) it is more pronounced among buffalo-derived parasites, as national parks and game ranches are fewer and more widely spaced, and translocation of wildlife occurs far less frequently than the movement of cattle. Furthermore, restriction of livestock movement by government policies such as Chapter 252 of the Laws of Zambia (the Stock Diseases Act), may further limit parasite transmission and recombination between populations. The LD and non-panmictic state of populations in this study may also have been due to the small sample size analysed, hence further studies would be necessary to confirm or annul the current findings. Nevertheless, the results are consistent with previous reports from Zambia on *T. parva* parasites associated with cattle, which were all in LD and non-panmictic state. The state of non-panmixia favours the strengthening of *T. parva* control efforts in Zambia, especially in the context of the use of ITM. A low number of MLGs may have implications for parasite control, as different genotypes may respond differently to interventions. This could require differentiated control strategies tailored to the specific genotypes present. A decrease in MLG numbers can also serve as evidence that interventions are effectively reducing parasite diversity, highlighting the importance of continuous monitoring to evaluate the long-term impact of control efforts. Notably, there was an exception of two populations which had more than one MLG; the cattle-derived parasite population from the Central Province with three MLGs and the buffalo-derived population from Southern Province with two. These observations suggest that there is some extent of genetic diversity in some *T. parva* populations from Zambia and this may be a developing phenomenon. Therefore, continuous surveillance is necessary to monitor and stay abreast of the dynamic nature of this parasite for effective control.

This study also showed that the minimum and maximum MOI were higher in parasite populations from buffalo compared to those from cattle, as well as in the combined parasite populations. A high MOI indicates that a host or population is infected with multiple distinct parasite genotypes. This is an important indicator of transmission intensity and can influence disease severity in some infections (Pacheco et al., 2016). The high MOI in buffalo-derived *T. parva* is expected since buffalo are reservoir hosts for this parasite. Oura et al. (2005) postulated that high MOI facilitates genetic exchange among *T. parva* populations. In *Plasmodium falciparum,* high MOI has also been associated with high asymptomatic prevalence, even in high exposure, creating a large reservoir of parasites that can be transmitted to others, posing a challenge for control interventions such as immunisation (Mwesigwa et al., 2024; Zhan et al., 2025). By inference, the high MOI in the buffalo-derived *T. parva* populations in Zambia may also pose challenges to the effectiveness of ITM, an intervention widely used in the control of *T. parva* infections in the country. Additionally, the PCoA for buffalo-derived parasite populations showed higher proportion of the total variance (6.67 %), compared to 2.97 and 3.9 % for the x- and y-axes coordinates for cattle populations, suggesting higher genetic diversity in sub-populations from the buffalo host.

The PCoA of genotypes from both cattle- and buffalo-derived *T. parva* showed limited genetic sub-clustering, with no geographic sub-clustering. This absence of geographic sub-structuring is contrary to findings of similar studies from other countries (Lubembe et al., 2020). However, the results from the current study are in congruence with a previous study examining *T. parva* populations from cattle in two Zambian provinces (Muleya et al., 2022), which suggests that *T. parva* populations in Zambia do not stratify according to geographic origin. The close genetic relationship among these populations and the lack of geographic sub-structuring may be attributed to the illegal movement of livestock, despite the enforcement of stock movement control regulations restricting unauthorized livestock movement in Zambia. A similar observation was made by a study on Foot-and-mouth diseases (FMD) by Banda et al. (2021) which demonstrated molecular based evidence of spread of FMD attributed to illegal movement of livestock. Strengthening enforcement of livestock movement regulations is therefore necessary to minimise transmission of the genetically diverse buffalo-derived *T. parva* parasites to cattle. Although genetic diversity was detected among genotypes from buffalo-derived parasites, some genotypes from the investigated sub-populations clustered together. The close genetic relationship between parasites from Central and Southern provinces is most likely due to the fact that buffalo populations in Central Province were sourced from Kafue National Park, part of which is situated in Southern Province.

Notably, several alleles were shared between the two vaccine strains, Katete and Chitongo, used for ITM in Zambia and the parasite populations from cattle and buffalo. Consistently, PCoA showed clustering of some vaccine genotypes with genotypes from these populations. This close relationship of vaccine genotypes with some of the buffalo-derived *T. parva* genotypes further suggests that the current vaccine may provide protection against some buffalo-derived genotypes. However, dedicated vaccine efficacy trials are needed to determine how efficacious the current ITM vaccines can be against infections with buffalo-derived parasites.

## 5 Conclusion

This study provides the first comprehensive analysis of the antigenic and genetic diversity of *T. parva* derived from buffalo in Zambia, in comparison with cattle-derived populations and the ITM vaccine stocks Katete and Chitongo. Both antigenic and genetic assessments revealed that buffalo-derived parasites are genetically distinct, although partially related to some cattle-derived populations and vaccine strains. Notably, buffalo-associated parasites exhibited greater antigenic and genetic diversity than those from cattle, indicating that the ITM vaccines currently deployed in Zambia may confer only limited protection against infections originating from buffalo reservoirs. Furthermore, evidence of non-panmictic population structures across all groups suggests restricted gene flow, despite the presence of shared genotypes. While these findings provide important insights into parasite diversity and its implications for vaccine efficacy, the limited sample size restricts broader generalization. Expanded studies with larger sample sets and wider geographic coverage, particularly in regions where buffalo and cattle populations interact, are required to confirm these observations.

## CRediT authorship contribution statement

**Chimvwele Namantala Choopa:** Writing – original draft, Investigation, Formal analysis. **Walter Muleya:** Writing – review & editing, Supervision, Formal analysis, Data curation. **Lubembe Donald Mukolwe:** Writing – review & editing, Formal analysis, Data curation. **Paul Fandamu:** Writing – review & editing. **Kgomotso Penelope Sibeko-Matjila:** Writing – review & editing, Visualization, Supervision, Project administration, Methodology, Funding acquisition, Data curation, Conceptualization.

## Funding

This study was supported under the National Research Foundation, South Africa, Competitive Programme for Rated Researchers (CPRR) Grant (grant number: 105982).

The authors are grateful to staff in the Department of Veterinary Services under the Ministry of Fisheries and Livestock, Zambia, for their assistance during cattle sample collection. We are also thankful to the Central Veterinary Research Institute for providing buffalo blood samples from its repository. We further extend our gratitude to the University of Pretoria, University of Zambia and Central Veterinary Research Institute for availing their laboratories for the project.

## Declaration of competing interest

The authors declare no conflict of interest.
