# Supplementary material for: Antigenic and genotypic relatedness of buffalo-derived Theileria parva from Zambia to cattle-derived parasites and vaccine stocks
Source: Int J Parasitol Parasites Wildl. 2025 Dec 10;29:101176. doi: 10.1016/j.ijppaw.2025.101176 (PMC12765116; doi:10.1016/j.ijppaw.2025.101176)
Supplement: Multimedia component 1 [file mmc1.docx]

## Supplementary Materials

Table S1: *Theileria parva* antigens Tp1 and Tp2 reference gene sequences, with accession numbers and the respective epitope regions

| **TpAg gene** | ***T. parva* reference ID/Name** | **Sequence accession number** | **Sample origin** | **CD8+ T-cell reference epitope(s)** | **Reference** |  |
| --- | --- | --- | --- | --- | --- | --- |
| Tp1 | Muguga | XM_757880 | Kenya | VGYPKVKEEML (Tp1_214-224_) | Graham *et al*., 2008 | |
|  | Kiambu 5 | JF451939 | Kenya | VGYPKVKEEML (Tp1_214-224_) | Pelle *et al*., 2011 | |
|  | Serengeti | JF451940 | Kenya | VGYPKVKEEML (Tp1_214-224_) |  |  |
|  | Chitongo | JF451975 | Zambia | VGYPKVKEEII (Tp1_214-224_) | Pelle et al., 2011 | |
|  | Katete KL 11 | LC645844 | Zambia | VGYPKVKEEML (Tp1_214-224_) | Muleya *et al*., 2022 | |
|  | 6C5 | LC645876 | Zambia | VGYPKVKEEII (Tp1_214-224_) |  |  |
|  | 89C58 | LC645939 | Zambia | VGYPKVKEEML (Tp1_214-224_) |  |  |
|  | 20BA89 | LC645961 | Zambia | VGYPKVKEEML (Tp1_214-224_) |  |  |
|  | 26BA95 | LC645967 | Zambia | VGYPKVKEEII (Tp1_214-224_) |  |  |
|  | Buffalo 5641 | JF452001 | Kenya | VGYPKVKEEML (Tp1_214-224_) | Pelle et al., 2011 | |
| Tp2 | Chitongo | JF451884 | Zambia | SDDELDTLGML (Tp2_27-37_) | Pelle *et al*., 2011 | |
|  | Muguga | JF451861 | Kenya | SHEELKKLGML (Tp2_27-37_) |  |  |
|  | Katete | JF451863 | Zambia | SHEELKKLGML (Tp2_27-37_) |  |  |
|  | Kiambu 5 | JF451880 | Kenya | SDEELNKLGML (Tp2_27-37_) |  |  |
|  | Serengeti | JF451862 | Kenya | SHEELKKLGML (Tp2_27-37_) |  |  |
|  | Buffalo 7344 | JF451899 | Kenya | SDEELESLGML (Tp2_27-37_) |  |  |
|  | 13BA60 | LC645835 | Zambia | SDNELDTLGLL (Tp2_27-37_) | Muleya *et al*., 2022 | |
|  | 22BA64 | LC645839 | Zambia | SHEELNKLGML (Tp2_27-37_) |  |  |
|  | 38C23 | LC645723 | Zambia | SHEELKKLGML (Tp2_27-37_) |  |  |
|  | 75C48 | LC645748 | Zambia | SHEELNKLGML (Tp2_27-37_) |  |  |
|  | 80C51 | LC645751 | Zambia | SHEELKKLGML (Tp2_27-37_) |  |  |
|  | 86C54 | LC645753 | Zambia | SEDELDTLGML (Tp2_27-37_) |  |  |
|  | 19C10 | LC645710 | Zambia | SDDELDTLGML (Tp2_27-37_) |  |  |

Table S2: Allele frequency ranges from Central, Eastern, Lusaka and Southern provinces.

| **Population** | **Allele frequency range per locus** | | | | | | | | | | | |
| --- | --- | --- | --- | --- | --- | --- | --- | --- | --- | --- | --- | --- |
|  | **MS7** | | **MS8** | | **MS19** | | **MS25** | | **MS33** | | **MS39** | |
|  | Cattle | Buffalo | Cattle | Buffalo | Cattle | Buffalo | Cattle | Buffalo | Cattle | Buffalo | Cattle | Buffalo |
| Central | 0.08 - 0.33 | 0.25 | 0.08 - 0.17 | 0.25 | 0.08 - 0.17 | 0.25 - 0.5 | 0.08 - 0.42 | 0.25 | 0.08 - 0.42 | 0.25 | 0.08 - 0.33 | 0.25 |
| Eastern | 0.17 - 0.5 | 0.17 | 0.17 - 0.5 | 0.17 | 0.17 - 0.33 | 0.17 - 0.5 | 1 | 1 | 0.17 - 0.5 | 0.17 | 0.17 | 0.17 |
| Lusaka | 0.06 - 0.44 | - | 0.06 - 0.19 | n/a | 0.06 - 0.38 | n/a | 0.06 - 0.31 | n/a | 0.06 - 0.38 | n/a | 0.06 - 0.5 | n/a |
| Southern | 0.09 - 0.27 | 0.25 | 0.09 - 0.27 | 0.25 | 0.09 - 0,27 | 0.5 | 0.09 - 0.27 | 0.25 | 0.09 - 0.56 | 0.25 | 0.09 - 0.5 | 0.25 |
| Katete | 1 | - | 1 | - | 1 | - | 1 | - | 1 | - | 1 | - |
| Chitongo | 1 | - | 1 | - | 1 | - | 1 | - | 1 | - | 1 | - |

n/a = not available; - = no samples analysed

Table S3: Alleles shared between populations from Central, Eastern, Lusaka and Southern provinces.

| **Populations** | **Common alleles per locus** | | | | | |
| --- | --- | --- | --- | --- | --- | --- |
|  | **MS7** | **MS8** | **MS19** | **MS25** | **MS33** | **MS39** |
| Cattle | n=5 | n=5 | n=8 | n=3 | n=5 | n=1 |
|  | 145 | 242 | 302 | 261 | 185 | 261 |
|  | 146 | **311** | 303 | 323 | 186 |  |
|  | 311 | 312 | 304 | 337 | 248 |  |
|  | 372 | 322 | 308 |  | 260 |  |
|  | ***309*** | 331 | 309 |  | 261 |  |
|  |  |  | 311 |  |  |  |
|  |  |  | 312 |  |  |  |
|  |  |  | 372 |  |  |  |
| Buffalo | n=0 | n=1 | n=2 | n=0 | n=0 | n=0 |
|  |  | 178 | 312 |  |  |  |
|  |  |  | 332 |  |  |  |
| Cattle *vs* buffalo | n=2 | n=3 | n=3 | n=2 | n=2 | n=2 |
|  | 311 | 148 | 303 | 240 | 220 | 245 |
|  | 372 | 242 | 311 | 336 | 309 | 295 |
|  |  | 292 | 312 |  |  |  |

Table S4: Predominant alleles detected from Central, Eastern, Lusaka and Southern provinces.

| **Population** | **Predominant alleles (bp) per locus** | | | | | | | | | | | |
| --- | --- | --- | --- | --- | --- | --- | --- | --- | --- | --- | --- | --- |
|  | **MS7** | | **MS8** | | **MS19** | | **MS25** | | **MS33** | | **MS39** | |
|  | Cattle | Buffalo | Cattle | Buffalo | Cattle | Buffalo | Cattle | Buffalo | Cattle | Buffalo | Cattle | Buffalo |
| Central | ***309*** | none | 302,  ***311***,  312 | none | 310 | **332** | **261** | none | **185** | none | 261, 323 | none |
| Eastern | 260 | none | 242 | none | 372 | 312 | 336 | 336 | **185** | none | none | none |
| Lusaka | 372 | - | ***311*** | - | **303** | - | **261** | - | **260** | - | 260 | - |
| Southern | ***309*** | none | 331 | none | **303** | 242, **332** | 245 | none | **260** | none | 245 | none |
| Katete | 146 | - | 231 | - | 268 | - | 201 | - | 248 | - | 377 | - |
| Chitongo | 146 | - | 143 | - | 268 | - | 200 | - | 146 | - | 201 | - |

- = no samples analysed, Bold = predominant alleles in multiple populations

Table S5: Linkage analysis of field parasites from Central, Eastern, Lusaka, and Southern Provinces of Zambia, together with the Chitongo and Katete vaccine stocks.

| Population | *I*_A_^S^ | *V*_D_ | *L_para_* | *L_MC_* | Linkage |
| --- | --- | --- | --- | --- | --- |
| Central cattle | 0.1716 | 2.7792 | 1.7035 | 1.9481 | LD |
| Central buffalo | 0.3302 | 3.3778 | 1.8117 | 2.0444 | LD |
| Central Province | 0.1117 | 2.2342 | 1.5731 | 1.7157 | LD |
| Eastern cattle | 0.3623 | 5.1000 | 2.3863 | 2.8000 | LD |
| Eastern buffalo | 0.2416 | 2.9905 | 1.6646 | 1.5905 | LD |
| Eastern Province | 0.1675 | 2.9565 | 1.7849 | 1.8137 | LD |
| Lusaka Province | 0.1411 | 2.5764 | 1.6417 | 1.6875 | LD |
| Southern cattle | 0.1987 | 3.5245 | 2.0985 | 2.3245 | LD |
| Southern buffalo | 0.1926 | 2.2778 | 1.6554 | 1.8333 | LD |
| Southern Province | 0.1578 | 2.9461 | 1.8131 | 1.8131 | LD |
| All cattle combined | 0.0536 | 2.0661 | 1.6715 | 1.7102 | LD |
| All buffalo combined | 0.0815 | 2.1288 | 1.6486 | 1.6288 | LD |
| All populations combined | 0.0453 | 1.9647 | 1.6100 | 1.6040 | LD |
| All combined + Vaccine | 0.0440 | 1.9504 | 1.6103 | 1.6191 | LD |
